# Bioactive Compounds in the Oils of the Autochthonous Slovenian Olive Varieties ‘Buga’, ‘Črnica’ and ‘Drobnica’

**DOI:** 10.3390/plants13141995

**Published:** 2024-07-22

**Authors:** Vasilij Valenčič, Milena Bučar-Miklavčič, Maja Podgornik

**Affiliations:** Science and Research Centre Koper, Garibaldijeva 1, 6000 Koper, Slovenia

**Keywords:** ‘Buga’, ‘Črnica’, ‘Drobnica’, chemistry, characterisation

## Abstract

The adaptation of autochthonous olive varieties to local soil and climatic conditions can lead to a unique chemical composition and characteristics of olive oils that may differ from the generally accepted quality standards set out in the International Olive Oil Council strategy documents and EU regulations. Therefore, the fatty acid composition, biophenol, tocopherol, sterol and triterpenic dialcohol content and composition of the autochthonous Slovenian olive varieties ‘Buga’, ‘Črnica’ and ‘Drobnica’ were studied for a three-year period with the aim of valorising the characteristics of the three olive varieties. Standardised and accredited analytical methods in accordance with SIST EN ISO/IEC 17025:2017 were applied. The results of the investigation showed that the highest average amount of oleic acid (75.75%) was found in the oils of the ‘Črnica’ variety, followed by the ‘Drobnica’ (72.06%) and the ‘Buga’ (68.73%). All three varieties are a good source of total biophenols (‘Buga’ 616 mg/kg, ‘Drobnica’ 569 mg/kg and ‘Črnica’ 427 mg/kg) and α-tocopherol (‘Buga’ 378 mg/kg, ‘Drobnica’ 279 mg/kg, and ‘Črnica’ 243 mg/kg). ‘Buga’ and ‘Drobnica’ are characterised by high amounts of total sterols, 2468 mg/kg and 2391 mg/kg, respectively, while ‘Črnica’ oils, in comparison, showed a lower average value of total sterols (1351 mg/kg). Due to their exceptional chemical composition, ‘Buga’, ‘Črnica’ and ‘Drobnica’ show great potential for the further cultivation and valorisation of traditional olive oil production in the region, thus contributing to the preservation of biodiversity and local traditions. The quality parameters of olive oil from the autochthonous Slovenian olive varieties ‘Buga’, ‘Črnica’ and ‘Drobnica’ also fulfil the limits for extra virgin olive oil according to the Commission Delegated Regulation (EU) 2022/2104, despite local climatic influences. However, accelerated growth due to climatic changes affecting early harvest can lead to them falling outside these limits, which was observed in particular for the ‘Buga’ variety in terms of the linoleic acid content. This study emphasises the importance of timing the harvest to achieve optimum maturity and meet EU quality standards, taking into account the genetic makeup of the varieties and their response to the current climatic conditions.

## 1. Introduction

The olive (*Olea europaea* L.) is one of the most important crops in Slovenia and is cultivated in the Primorska region, which is characterised by a Mediterranean climate. The last edition of Fruit Selection for Slovenia [1] reported that based on the number of cultivated hectares, the olive tree is the second most common culture among the fruit species in Slovenia, despite the limited possibilities of spreading. Official data for the year 2023 [2] shows that there are 2571 ha of olive orchards in Slovenia. Most of them are located in the region of Slovenian Istria (96%), while the remainder (4%) are found in the regions of the Goriška Brda, the Vipava Vally and the Karst. Slovenia is one of the most northerly regions of the Mediterranean where olives are still cultivated. Due to climate change and new climatic conditions, the northern Mediterranean countries could now become important oil producers. Tanasaijevic et al. [3] have shown, based on regional climate models driven by ECHAM5 for the A1B scenario of the Special Report on Emission Scenarios (SRES) and the agro-ecological zoning method, that the potentially cultivable areas for olive growing are expected to expand northwards and to higher altitudes, increasing by 25% in 50 years. In Slovenia, the average yield of olives is 2.5 t/ha and average olive oil production is 400 kg/ha, with a total average annual production of 700 t of extra virgin olive oil. Despite the relatively low production levels, the geographical area of Slovenian Istria enables the production of high-quality extra virgin olive oil.

In the northern part of the Adriatic region, the most widespread olive variety is the ‘Istrska Belica’, which is cultivated intensively in the regions of the Slovenian Istria and the Friuli Venezia Giulia (Italy). According to oral tradition, the ‘Istrska Belica’ variety began to spread through Slovenian Istria after the severe frost of 1929, as it is resistant to low temperatures, produces good and regular yields and has a high oil content [4]. Before the ‘Istrska Belica’, the varieties ‘Buga’ (‘Burla’), ‘Drobnica’ (‘Komuna’), ‘Črnica’ (‘Carbon’), ‘Štorta’, ‘Mata’, ‘Žižula’ and ‘Zmartel’ were the most widespread olive varieties in the area, being replaced by new, higher-yielding varieties due to their low and alternative yields [5]. This led to accelerated genetic erosion, the abandonment of traditional olive groves and centuries-old olive trees, overgrowth of the cultivated landscape and the establishment of intensive, economically profitable plantations. Nowadays, however, the production of monovarietal oils obtained from a specific local autochthonous variety with specific characteristics emphasising its identity and uniqueness is being promoted [6]. This leads to the preservation of autochthonous and traditional varieties of agricultural plants and protects genetic diversity, enabling better adaptation to new and rapidly changing environmental conditions, which is a prerequisite for survival in a given climatic environment.

The adaptation of autochthonous olive varieties to local soil and climatic conditions can lead to a unique chemical composition and characteristics of the olive oils, which may differ from the generally accepted quality standards and purity characteristics set out in the International Olive Oil Council strategy documents and the Commission Delegated Regulation (EU) 2022/2104 [7]. For example, autochthonous varieties may contain different levels of tocopherols (vitamin E) and biophenols, which act as natural antioxidants and help prevent a range of diseases and benefit human health. Understanding these specific characteristics is crucial for the adaptation of the quality parameters in EU legislation. Recognising variations as a specificity of varieties is crucial for the preservation of their cultural and economic value and gives consumers access to a wide range of high-quality olive oils.

Therefore, the aim of this study was to systematically analyse the main bioactive compounds of ‘Buga’, ‘Črnica’ and ‘Drobnica’ olive oil during a three-year period from 2018 to 2020. The acquired data are very important for valorising the characteristics of the autochthonous varieties that are typical of the territory, can certify the protected designation of origin, and evaluate the compliance of the produced oils with international standards. These include the trade standards applying to olive oils and olive pomace oils released by the International Olive Council [8] and the EU legislation on olive oil, the Commission Delegated Regulation (EU) 2022/2104 [7].

## 2. Results

### 2.1. Fatty Acid Composition

The fatty acid composition was determined in olive oils produced from the ‘Buga’, ‘Črnica’ and ‘Drobnica’ varieties. The results of the means and the standard deviations are presented in Table 1. Statistically significant differences (*p* < 0.05) between the three olive varieties were found for most of the fatty acids, though there were no significant differences between the ‘Buga’, ‘Črnica’ and ‘Drobnica’ varieties for myristic (C 14:0), heptadecenoic (C 17:1) and lignoceric (C 24:0) acids.

The LSD multiple comparisons test showed statistically significant differences (*p* < 0.05) in the average content of palmitic (C 16:0), stearic (C 18:0), oleic (C 18:1) and behenic (C 22:0) acids between the cultivars of ‘Buga’ and ‘Črnica’, ‘Buga’ and ‘Drobnica’, and ‘Črnica’ and ‘Drobnica’. The highest average content of palmitic acid was determined in samples of the ‘Buga’ variety (16.45%), followed by the ‘Drobnica’ (14.52%) and ‘Črnica’ (14.08%) varieties. The ‘Črnica’ variety showed the highest average amount of oleic (75.75%) and stearic (2.12%) acids, followed by the ‘Drobnica’ (72.06% oleic acid and 1.97% stearic acid) and ‘Buga’ (68.73% oleic acid and 1.60% stearic acid). The differences in behenic acid content were less pronounced between the three varieties: 0.12% in ‘Drobnica’, 0.11% in ‘Črnica’ and 0.10% in ‘Buga’ samples.

Statistically significant differences (*p* < 0.05) between the ‘Buga’ and ‘Črnica’, and the ‘Buga’ and ‘Drobnica’ were determined in the case of palmitoleic (C 16:1), heptadecanoic (C 17:0), linolenic (C 18:3) and arachidic (C 20:0) acids, whereas there were no significant differences for the studied compounds between the ‘Črnica’ and ‘Drobnica’. The olive oil samples of ‘Buga’, compared to ‘Črnica’ and ‘Drobnica’, showed the highest amounts of palmitoleic (2.84%) and linolenic (0.92%) acids, and the lowest amounts of heptadecanoic (0.03%) and arachidic (0.32%) acids, although the differences for heptadecanoic and arachidic acids are minimal between the three varieties.

Statistically significant differences (*p* < 0.05) between the ‘Buga’ and ‘Črnica’, and the ‘Črnica’ and ‘Drobnica’ were determined for linoleic acid (C 18:2), whereas there were no significant differences between the ‘Buga’ and ‘Drobnica’. The lowest average amount of linoleic acid (4.54%) was determined in the ‘Črnica’ samples, while the ‘Drobnica’ and ‘Buga’ samples showed a higher content of linoleic acid, 7.87% and 8.58%, respectively.

Statistically significant differences (*p* < 0.05) between the ‘Buga’ and ‘Drobnica’, and the ‘Črnica’ and ‘Drobnica’ were determined for eicosenoic acid (C 20:1), whereas there were no significant differences between the ‘Buga’ and ‘Črnica’. The highest amount of eicosenoic acid (0.33%) was determined in the ‘Drobnica’ samples, followed by the ‘Buga’ (0.28%) and ‘Črnica’ (0.27%), as is shown in Table 1.

The total saturated fatty acids (SFA), total monounsaturated fatty acids (MUFA), total polyunsaturated fatty acids (PUFA), PUFA/SFA ratio and oleic acid/linoleic acid ratio were calculated. Statistically significant differences (*p* < 0.05) between the studied olive varieties were determined for all these parameters. The ‘Črnica’ showed the highest value of the MUFA, the lowest values of the SFA and PUFA, and the highest ratio between the PUFA and SFA and between the oleic and linoleic acids.

### 2.2. Biophenol and Tocopherol Content and Composition

The results of the determination of biophenols and tocopherols in the samples of the ‘Buga’, ‘Črnica’ and ‘Drobnica’ varieties are shown in Table 2. Statistically significant differences (*p* < 0.05) between the three olive varieties were determined for the total ligstroside biophenols and lignans. The ‘Buga’ olive variety is characterised by the highest average amount of total ligstroside biophenols (188 mg/kg), followed by the ‘Črnica’ (156 mg/kg) and the ‘Drobnica’ (125 mg/kg). The ‘Črnica’ variety shows the highest content of lignans (83 mg/kg), compared to the ‘Buga’ (26 mg/kg) and the ‘Drobnica’ (19 mg/kg).

Statistically significant differences (*p* < 0.05) between the ‘Buga’ and ‘Črnica’, and the ‘Črnica’ and ‘Drobnica’ were determined in the case of the total oleuropein biophenols, total biophenols and the following oleuropein derivatives: the dialdehydic form of decarboxymethyl oleuropein aglycone (DMO-Agl-dA), the dialdehydic form of oleuropein aglycone (O-Agl-dA), and the aldehydic form of oleuropein aglycone (O-Agl-A). Whereas, there were no significant differences between the ‘Buga’ and ‘Drobnica’ in the biophenol compounds. The results presented in Table 2 show that the ‘Buga’ and ‘Drobnica’ have the highest average amounts of total oleuropein biophenols (‘Buga’ 373 mg/kg and ‘Drobnica’ 403 mg/kg) and total biophenols (‘Buga’ 616 mg/kg and ‘Drobnica’ 569 mg/kg), compared to the ‘Črnica’ (170 mg/kg of total oleuropein biophenols and 427 mg/kg of total biophenols). The ‘Buga’ and ‘Drobnica’ are also characterised by higher amounts of DMO-Agl-dA, O-Agl-dA and O-Agl-A compared to the ‘Črnica’, as shown in Table 2.

The ‘Drobnica’ olive variety has the lowest average content (23 mg/kg) of the dialdehydic form of decarboxymethyl ligstroside aglycone (DML-Agl-dA) and it is statistically different (*p* < 0.05) from the ‘Buga’ (47 mg/kg) and ‘Črnica’ (55 mg/kg). The highest amount of the dialdehydic form of ligstroside aglycone (L-Agl-dA) was determined for the ‘Buga’ variety (31 mg/kg), followed by the ‘Drobnica’ (21 mg/kg) and ‘Črnica’ (15 mg/kg), whereas the aldehydic form of ligstroside aglycone (L-Agl-A) varied from 7 mg/kg in the ‘Črnica’ to 10 mg/kg in the ‘Drobnica’ and 11 mg/kg in the ‘Buga’ olive oil samples (*p* < 0.05).

Statistically significant differences (*p* < 0.05) between the three studied olive varieties were determined in the content of α- and γ-tocopherol. The LSD multiple comparisons test showed statistically significant differences (*p* < 0.05) between the ‘Buga’ and ‘Črnica’, the ‘Buga’ and ‘Drobnica’, and the ‘Črnica’ and ‘Drobnica’ in the content of both tocopherol isomers. The highest average content of α-tocopherol was determined in the samples of the ‘Buga’ variety (378 mg/kg), followed by the ‘Drobnica’ (279 mg/kg) and ‘Črnica’ (243 mg/kg). All three varieties showed a lower content of γ-tocopherol compared to α-tocopherol. The results showed 14 mg/kg of γ-tocopherol in the ‘Buga’, 8 mg/kg in the ‘Drobnica’ and 4 mg/kg in the ‘Črnica’. β- and δ-tocopherols were not detected in any samples of the three varieties (below the limit of detection of 3 mg/kg).

### 2.3. Sterols and Triterpenic Dialcohols

The results of the determination of sterols, triterpenic dialcohols erythrodiol and uvaol are presented in Table 3. Statistically significant differences (*p* < 0.05) between the ‘Buga’, ‘Črnica’ and ‘Drobnica’ were determined in the content of sitostanol, Δ-5-avenasterol, Δ-5,24-stigmastadienol and Δ-7-avenasterol. The ‘Drobnica’ olive variety has the highest content of Δ-5-avenasterol (11.72%), Δ-5,24-stigmastadienol (1.26%) and Δ-7-avenasterol (0.68%), compared to the other two varieties, while a higher value of sitostanol (3.51%) was present in the ‘Črnica’ samples. The ‘Drobnica’ variety is also statistically different from the ‘Buga’ and ‘Črnica’ in the clerosterol and β-sitosterol content. In the case of clerosterol, there were minor differences (from 0.99% to 1.06%) between the olive varieties, whereas for β-sitosterol, the most abundant sterol, higher values were determined in the ‘Buga’ (84.67%) and ‘Črnica’ (85.23%) samples, than in the ‘Drobnica’ (79.45%).

The ‘Črnica’ olive variety is statistically different (*p* < 0.05) from the ‘Buga’ and ‘Drobnica’ in the content of cholesterol, 24-methylene-cholesterol, campesterol, campestanol, Δ-7-stigmastenol and total sterols. The ‘Črnica’ variety is characterised by higher values of cholesterol (0.13%), campesterol (3.70%), campestanol (0.34%), Δ-7-stigmastenol (0.29%) and lower values of 24-methylene-cholesterol (0.08%) and total sterols (1351 mg/kg), compared to the ‘Buga’ and ‘Drobnica’.

There were no statistically significant differences between the three studied varieties in the content of stigmasterol and apparent β-sitosterol, whereas the ‘Črnica’ showed a higher content of stigmasterol (1.00%) and a lower content of apparent β-sitosterol (93.98%) compared to the ‘Buga’ (stigmasterol 0.78%, apparent β-sitosterol 95.56%) and the ‘Drobnica’ (stigmasterol 0.75%, apparent β-sitosterol 95.35%).

The ‘Črnica’ and ‘Drobnica’ varieties showed statistically significant differences (*p* < 0.05) in the content of triterpenic dialcohols erythrodiol and uvaol, which varied from 0.66% in ‘Črnica’ to 1.06% in ‘Drobnica’ samples.

## 3. Discussion

### 3.1. Fatty Acid Composition

According to the methodology for secondary characterisation by the International Olive Council [9] and considering the variation of the data, the ‘Črnica’ and ‘Drobnica’ olive varieties are characterised by a high oleic acid content (C 18:1), 75.75% and 72.06%, respectively, whereas the ‘Buga’ variety has a medium (68.73%) oleic acid content. The opposite situation was observed in the case of linoleic acid (C 18:2): ‘Črnica’ has a very low (4.54%) linoleic acid content, and ‘Drobnica’ (7.87%) and ‘Buga’ (8.58%) have low linoleic acid contents, which, consequently, affects the ratio between oleic and linoleic acids. The ‘Črnica’ showed the highest oleic/linoleic ratio (16.99), followed by ‘Drobnica’ (9.67) and then ‘Buga’ (8.32), which indicates that the oleic/linoleic acid ratio is influenced by the olive variety. Our results are in accordance with Hernández et al. [10], who stated that oleic and linoleic acid contents displayed the highest degree of variability of the different fatty acids present in olive oils. In addition, Brkić-Bubola et al. [11] reported a high oleic/linoleic ratio in the Croatian ‘Rosinjola’ and ‘Istarska bjelica’ oils, which indicated their higher oxidative stability compared to other investigated oils. It was found that the content of oleic acid in the ‘Buga’ variety is in the same range as the Croatian ‘Buža’ (67.73%), ‘Buža puntoža’ (67.81%) and ‘Bova’ (64.51%) varieties, as reported by Brkić-Bubola et al. (2018), whereas the content of oleic acid in the ‘Črnica’ and ‘Drobnica’ is in the same range as the Slovenian ‘Istrska belica’ (75.35%) and ‘Leccino’ (72.74%) as reported by Bešter et al. [12], and the Croatian ‘Istarska bjelica’ (73.93%) and ‘Rosinjola’ (74.82%). The palmitic acid content (C 16:0) is also interesting, as the extra virgin olive oils from the ‘Buga’ have a very high palmitic acid content (16.45%), while the oils from the ‘Drobnica’ (14.52%) and ‘Črnica’ (14.08%) have a high content of C 16:0, which is consequently related to the synthesis and content of oleic acid, since palmitate is the precursor of oleic acid and longer-chain fatty acids formed by the elongation and desaturation biosynthesis of fatty acids [13,14].

The fatty acid composition of the three olive varieties is in accordance with the limit values for extra virgin olive oils set in the Commission Delegated Regulation (EU) 2022/2104 [7]. The ‘Buga’ variety shows a high average value (0.92%) of linolenic acid (C 18:3), which in some cases, usually at the end of September, can reach the upper border of the limit value set in the legislation, i.e., ≤1.0% (Commission Delegated Regulation (EU) 2022/2104) [7]. Nevertheless, it is still in accordance with the latest revision of the International Olive Council’s trade standards for olive oils and olive pomace oils [8], which prescribes that a virgin olive oil with a linolenic acid content that lies between 1.00% and 1.40% is considered authentic if the ratio between the apparent β-sitosterol and campesterol is greater than or equal to 24 and all the other purity criteria lie within the official limits. The ratio between the apparent β-sitosterol and campesterol can be calculated from the research data, and in the case of the ‘Buga’ it is 37.5, ‘Črnica’ is 25.4, and ‘Drobnica’ is 36.7. The other purity parameters were not determined, because that was not the aim of this work and the traceability from the olive fruits to the produced oils is guaranteed in this case, as the olive samples were manually picked by the laboratory staff and all the production processes were under control in the laboratory olive mill.

The ‘Črnica’ extra virgin olive oils are very interesting due to their potential to be included in the certification of the protected designation of origin (PDO) ‘Ekstra deviško oljčno olje Slovenske Istre’, as they are characterised by a high level of oleic acid (75.75%) and a low level of linolic acid (4.54%). They also fall within the limits set in the ‘Specification of PDO Ekstra deviško oljčno olje Slovenske Istre’ [15], which prescribes a minimum of 72% oleic acid and a maximum of 8% linolic acid. The use of ‘Drobnica’ oils for the PDO is limited and mainly depends on the other varieties present in the mixture, which can affect the fatty acid composition of the prepared oil and consequently the content of oleic and linoleic acids. Due to the low oleic acid content and high linoleic acid content, the use of ‘Buga’ oils is not recommended for the PDO, although we need to consider that this variety has a high total biophenols content (616 mg/kg) and produces good quality olive oils that can be successfully present on the market.

### 3.2. Biophenol and Tocopherol Content and Composition

The ‘Buga’ and ‘Drobnica’ olive varieties have high total biophenols content, 616 mg/kg and 569 mg/kg, respectively, while the ‘Črnica’ variety is among the oils with a medium (427 mg/kg) total biophenols content according to the methodology for secondary characterisation by the International Olive Council [9]. In Slovenia, the ‘Istrska belica’ variety with 598 mg/kg of total biophenols and the ‘Leccino’ variety with 399 mg/kg of total biophenols are also present [12]. A high total biophenols content was also determined in the Croatian ‘Buža’ variety (511.5 mg/kg), as reported by Novoselic et al. [16], and the ‘Oblica’ (from 537 mg/kg to 788 mg/kg, depending on the degree of ripening) as reported by Lukić et al. [17].

In the ‘Buga’ and ‘Drobnica’ oils, the total oleuropein biophenol levels are predominant (373 mg/kg and 403 mg/kg, respectively), followed by the total ligstroside biophenols (188 mg/kg and 125 mg/kg, respectively). The difference between the total oleuropein and total ligstroside compounds are less expressed in the ‘Črnica’ variety (170 mg/kg of the total oleuropein biophenols and 156 mg/kg of the total ligstroside biophenols). In general, all three studied varieties are characterised by the predominance of oleuropein derivatives in comparison to ligstroside derivatives, and between them, the most present is the dialdehydic form of decarboxymethyl aglycone derivatives, followed by the dialdehydic and aldehydic forms of oleuropein and ligstroside aglycones.

In relation to the ‘Specification for the Ekstra deviško oljčno olje Slovenske Istre’ with PDO, the Slovenian ‘Buga’, ‘Črnica’ and ‘Drobnica’ varieties show appropriate biophenol content, as the minimum prescribed amount of total biophenols is 150 mg/kg; however, it is also necessary to take into account the restrictions regarding the fatty acid composition, as already stated in the previous chapter.

Considering the average amount of tocopherols and the variation in the data, the ‘Buga’ variety is characterised by a high level of α-tocopherol and total tocopherols (sum of α- and γ-tocopherols), whereas the ‘Črnica’ and ‘Drobnica’ have a medium tocopherols content. Tocopherol content can be influenced by the olive variety and crop year, as is shown in the literature data [18] on the neighbouring Croatian ‘Buža’ (228–260 mg/kg), ‘Leccino’ (241–389 mg/kg) and ‘Rosulja’ varieties (211–351 mg/kg), which showed low to middle tocopherols contents for the years 2010 and 2011. The tocopherols contents can be affected by several factors; for example, Borges et al. [19], who studied the ‘Arbequina’ variety in Spain and Brazil, reported that climatic and geographic factors in the production zones seem to significantly affect the contents of tocopherols and biophenols. The ‘Buga’, ‘Črnica’ and ‘Drobnica’ varieties are a good source of tocopherols, which also contribute to the oxidative stability of olive oils. This finding is supported by Franco et al. [20], who reported a high positive correlation with oxidative stability for seven Spanish olive varieties.

### 3.3. Sterols and Triterpenic Dialcohols

The sterol content and composition of the three olive varieties are in accordance with the limit values for extra virgin olive oils as set in the Commission Delegated Regulation (EU) 2022/2104 [7] and the IOC trade standards for olive oils and olive pomace oils [8]. The ‘Buga’ and ‘Drobnica’ are characterised by large amounts of total sterols, 2468 mg/kg and 2391 mg/kg, respectively, while ‘Črnica’ oils, in comparison, showed a lower average value of total sterols (1351 mg/kg), and are more similar to ‘Istrska belica’, the most widespread variety in Slovenia, which has 1265 mg/kg of total sterols [21]. Compared to the ‘Buga’ and ‘Drobnica’, the ‘Črnica’ variety also has higher average values of campesterol (3.70%) and Δ-7-stigmastenol (0.29%). Regarding sterol composition, β-sitosterol, Δ-5-avenasterol and campesterol were the predominant sterols for all three studied varieties, which is in accordance with Yorulmaz et al. [22]. The β-sitosterol contents of the ‘Buga’ and ‘Drobnica’ varieties are comparable to the Croatian ‘Bova’ (87.89%), ‘Buža’ (87.51%), ‘Buža puntoža’ (84.08%) and ‘Rosinjola’ varieties (82.05%), as reported by Brkić-Bubola et al. [11], which have antioxidant effects on human tissues [23]. The ‘Buga’ variety shows 7.94% Δ-5-avenasterol and is comparable, considering the variation of the data, to the Croatian ‘Buža’ (6.18%) and ‘Buža puntoža’ (9.97%), while ‘Drobnica’ (11.72%) is comparable to ‘Rosinjola’ (10.61%). The campesterol content of ‘Buga’ (2.55%) and ‘Drobnica’ (2.60%) is in the same range of the Croatian ‘Buža’ (2.87%).

Brassicasterol, Δ-7-campesterol and Δ-5,23-stigmastadienol were not detected in any of the samples, while the triterpenic dialcohols erythrodiol and uvaol were slightly higher in the ‘Drobnica’ variety (1.06%) compared to the ‘Buga’ (0.79%) and ‘Črnica’ (0.66%), though far from the limit value of 4.5% as set in the Commission Delegated Regulation (EU) 2022/2104 [7].

## 4. Materials and Methods

### 4.1. Material

Olive fruits of the ‘Buga’, ‘Črnica’ and ‘Drobnica’ varieties were collected at 3 locations: Purissima (a collection plantation established between 2004 and 2006 in the Slovenian Istra), Sečovlje (a traditional productive plantation established before 1929 in the Slovenian Istra) and Šempeter (a collection plantation established between 2007 and 2014 in the Vipava Valley). This occurred in 2018, 2019 and 2020 during three periods from 20 September to 5 November (early maturity in the 38th, mid-maturity in the 41st and late maturity in the 44th week in each year) based on the ripeness stage. For each sample, approximately 1 kg of olive fruits were manually collected. The olive oils were produced using an Abencor system MC2 laboratory olive mill (MC2 Ingenieria y Sistemas, Sevilla, Spain).

### 4.2. Methods

All the methods used for determining the fatty acids, biophenols, tocopherols, sterols and triterpenic dialcohols were accredited in accordance with SIST EN ISO/IEC 17025:2017 [24]. All the chemicals reported in the following subsections met the requirements of the official methods and were purchased from Sigma–Aldrich Chemie GmbH (Munich, Germany).

#### 4.2.1. Determination of Fatty Acids

Fatty acids were determined in accordance with the Commission Regulation (EEC) No 2568/91, Annex X [25]. Fatty acid methyl esters were prepared in heptane with a 2-M methanolic potassium hydroxide solution and determined by gas chromatography. An Agilent HP 6890 Series (Agilent Technologies, Santa Clara, CA, USA), equipped with Supelco 2560 Capillary GC Column (100 m × 0.25 mm ID, df 0.20 μm; Supelco Inc., Bellefonte, PA, USA) and FID detector was used. Fatty acids were assigned by comparing the retention times with those of the reference standard Supelco 37 Component FAME Mix.

#### 4.2.2. Determination of Biophenols

Biophenols were determined in accordance with the method of the International Olive Council (IOC), COI/T.20/Doc. No 29/Rev. 1 [26]. The extraction was performed from 2.0 g of oil with the addition of 1 mL of internal standard solution (syringic acid 0.15 mg/mL). The sample was shaken with the aid of a Vortex-genie 2 G-560 E agitator (Scientific Industries Inc., Bohemia, NY, USA) for 30 s, then 5 mL of methanol/water 80/20 (*v*/*v*) extraction solution was added and shaken for 1 min. Biophenols were extracted using the ultrasonic bath ELMA D-78244 (ELMA, Singen, Germany) for 15 min at room temperature, followed by centrifugation with the aid of the Eppendorf centrifuge 5430R (Eppendorf SE, Hamburg, Germany) at 5000 rpm for 25 min. An aliquot of the supernatant phase was filtered through a 5 mL plastic syringe with a 0.45 µm PVDF filter in a 2 mL vial. Biophenols were determined using HPLC analysis as set out in the IOC method. An Agilent 1200 Series HPLC System (Agilent Technologies, Santa Clara, CA, USA) equipped with a binary pump and automatic liquid sampler, C18 reversed-phase column (Phenomenex synergi hydro, 250 × 4.6 mm, 4 µm; Phenomemex, Inc., Torrance, CA, USA), operating at 20 °C, with DAD detection at 280 nm was used. Spectral data for the peaks were recorded in the range of 200–600 nm. The mobile phase used was a gradient consisting of 0.2% aqueous H_3_PO_4_ (by volume) (A) and methanol/acetonitrile 1/1 (by volume) (B). The initial gradient composition was A at 96% and B at 4%. After forty min, the ratio of B increased to 50%, then to 60% in the next five min, and to 100% in the last fifteen min. After 72 min, the concentration of B was put at an initial value of 4%. The column was then equilibrated for 10 min before the next injection. A volume of 10 μL of the methanolic extract was injected into the system; the flow rate was 1 mL/min. An external calibration solution of tyrosol (0.030 mg/mL) and syringic acid (0.015 mg/mL) was prepared. All the biophenol compounds were quantified using the response factor for tyrosol and assigned by comparing their relative retention times to the retention time of the internal standard syringic acid.

#### 4.2.3. Determination of Tocopherols

Tocopherols were determined in accordance with SIST EN ISO 9936:2016 [27]. 100 mg of oil was weighed into a 10 mL volumetric flask and dissolved with n-heptane. An Agilent 1100 Series HPLC System (Agilent Technologies, Santa Clara, CA, USA) equipped with a binary pump and automatic liquid sampler, a C18 column (Phenomenex Luna 5μ Silica (2), 250 × 4.6 mm, 5μ; Phenomemex, Inc., Torrance, CA, USA), operating at 25 °C, with a fluorescence detector with the excitation wavelength set at 290 nm and the emission wavelength at 330 nm was used. The mobile phase used was n-heptane: tetrahydrofuran (96.2: 3.8, by volume) at a flow rate of 1.0 mL/min. The α-, β-, γ- and δ-tocopherol were determined. The method is validated from 3 to 2220 mg/kg.

#### 4.2.4. Determination of Sterols and Triterpenic Dialcohols

Sterols and triterpenic dialcohols were determined in accordance with the Commission Regulation (EEC) No 2568/91, Annex XIX [28], and were extracted from 5.0 g of oil in the presence of the internal standard α-cholestanol (0.2%, *m*/*v*). The sample preparation involved saponification with 2-M ethanolic potassium hydroxide solution, solvent extraction of unsaponifiable matter with diethyl ether, the separation of sterol and triterpenic dialcohols from the unsaponifiable matter with thin-layer chromatography, derivatisation into trimethylsilyl ethers, and determination using gas chromatography. An Agilent HP 6890 Series (Agilent Technologies, Santa Clara, CA, USA), equipped with a Supelco SPB-5 Capillary GC Column (60 m × 0.53 mm ID, df 5.00 μm; Supelco Inc., Bellefonte, PA, USA) and FID detector was used. The sterols and triterpenic dialcohols were assigned by comparing their relative retention times to the retention time of the internal standard α-cholestanol.

#### 4.2.5. Statistical Analysis

All the data were statistically analysed and expressed as mean values ± standard deviations. The significance of the difference was analysed using an ANOVA for equality of means, and a post-hoc Fisher’s least significant difference (LSD) test to find out the differences between each group was performed using SPSS Statistics v. 26 (SPSS, Chicago, IL, USA), with the statistical significance set at *p* < 0.05.

## 5. Conclusions

The extra virgin olive oils of the autochthonous ‘Buga’, ‘Črnica’ and ‘Drobnica’ varieties are characterised by medium to high oleic acid, tocopherols and biophenols contents and a low linoleic acid content. The varieties are well-adapted to the climatic conditions of the local environment and are a good source of natural antioxidants (biophenols and tocopherols), monounsaturated fatty acids, and sterols. Due to their exceptional chemical composition, the ‘Buga’, ‘Črnica’ and ‘Drobnica’ show great potential for further cultivation and the valorisation of traditional olive oil production in the region, thus contributing to the preservation of biodiversity and local traditions. In particular, the ‘Črnica’ variety has sufficient chemical characteristics to be included in the PDO certification ‘Ekstra deviško oljčno olje Slovenske Istre’ [15], while the other two varieties could be included with restrictions, which could possibly contribute to greater visibility of the oils of the region. The quality parameters of olive oil from the autochthonous Slovenian ‘Buga’, ‘Črnica’ and ‘Drobnica’ olive varieties also fulfil the limits for extra virgin olive oil according to the Commission Delegated Regulation (EU) 2022/2104, despite local climatic influences. However, accelerated growth due to climatic changes affecting the early harvest can lead them to fall below these limits, which was observed in particular for the ‘Buga’ variety for the linoleic acid content. This study emphasises the importance of timing the harvest to achieve optimum maturity and meet the EU quality standards, taking into account the genetic makeup of the varieties and their responses to current climatic conditions.

## Figures and Tables

**Table 1:** Fatty acid composition of the ‘Buga’, ‘Črnica’ and ‘Drobnica’ olive oils.

Fatty Acid	Unit	‘Buga’	‘Črnica’	‘Drobnica’	Purity Characteristics for Extra Virgin Olive Oil [7]
Myristic acid (C 14:0)	%	0.01 ± 0.001 ^a^	0.01 ± 0.001 ^a^	0.01 ± 0.001 ^a^	≤0.03
Palmitic acid (C 16:0)	%	16.45 ± 0.62 ^a^	14.08 ± 0.75 ^b^	14.52 ± 0.75 ^c^	7.00–20.00
Palmitoleic acid (C 16:1)	%	2.84 ± 0.44 ^a^	1.77 ± 0.36 ^b^	1.77 ± 0.27 ^b^	0.30–3.50
Heptadecanoic acid (C 17:0)	%	0.03 ± 0.01 ^a^	0.04 ± 0.01 ^b^	0.04 ± 0.01 ^b^	≤0.40
Heptadecenoic acid (C 17:1)	%	0.08 ± 0.01 ^a^	0.08 ± 0.01 ^a^	0.08 ± 0.01 ^a^	≤0.60
Stearic acid (C 18:0)	%	1.60 ± 0.11 ^a^	2.12 ± 0.14 ^b^	1.97 ± 0.13 ^c^	0.50–5.00
Oleic acid (C 18:1)	%	68.73 ± 2.34 ^a^	75.75 ± 1.04 ^b^	72.06 ± 2.48 ^c^	55.00–85.00
Linoleic acid (C 18:2)	%	8.58 ± 1.58 ^a^	4.54 ± 0.64 ^b^	7.87 ± 1.87 ^a^	2.50–21.00
Linolenic acid (C 18:3)	%	0.92 ± 0.11 ^a^	0.79 ± 0.14 ^b^	0.78 ± 0.10 ^b^	≤1.00
Arachidic acid (C 20:0)	%	0.32 ± 0.01 ^a^	0.38 ± 0.04 ^b^	0.39 ± 0.03 ^b^	≤0.60
Eicosenoic acid (C 20:1)	%	0.28 ± 0.02 ^a^	0.27 ± 0.03 ^a^	0.33 ± 0.02 ^b^	≤0.50
Behenic acid (C 22:0)	%	0.10 ± 0.01 ^a^	0.11 ± 0.02 ^b^	0.12 ± 0.01 ^c^	≤0.20
Lignoceric acid (C 24:0)	%	0.06 ± 0.01 ^a^	0.06 ± 0.01 ^a^	0.07 ± 0.01 ^a^	≤0.20
SFA	%	18.57 ± 0.64 ^a^	16.80 ± 0.84 ^b^	17.11 ± 0.76 ^c^	
MUFA	%	71.92 ± 2.02 ^a^	77.87 ± 0.89 ^b^	74.25 ± 2.33 ^c^	
PUFA	%	9.50 ± 1.58 ^a^	5.33 ± 0.55 ^b^	8.65 ± 1.83 ^c^	
PUFA/SFA ratio	-	4.39 ± 0.18 ^a^	4.97 ± 0.30 ^b^	4.86 ± 0.26 ^c^	
Oleic acid/linoleic acid ratio	-	8.32 ± 1.75 ^a^	16.99 ± 2.36 ^b^	9.67 ± 2.34 ^c^	

Abbreviations: SFA: saturated fatty acids; MUFA: monounsaturated fatty acids, PUFA: polyunsaturated fatty acids. The data are expressed as means ± standard deviations; *n* = 70; values in the same row with different superscript letters are statistically significantly different (*p* < 0.05).

**Table 2:** Biophenol and tocopherol content (mg/kg) and the composition of the ‘Buga’, ‘Črnica’ and ‘Drobnica’ olive oils.

Biophenols and Tocopherols (mg/kg)	‘Buga’	‘Črnica’	‘Drobnica’
Total biophenols	616 ± 152 ^a^	427 ± 113 ^b^	569 ± 193 ^a^
Total oleuropein biophenols	373 ± 103 ^a^	170 ± 64 ^b^	403 ± 167 ^a^
Total ligstroside biophenols	188 ± 40 ^a^	156 ± 54 ^b^	125 ± 31 ^c^
Lignans	26 ± 12 ^a^	83 ± 14 ^b^	19 ± 9 ^c^
DMO-Agl-dA	190 ± 52 ^a^	110 ± 38 ^b^	180 ± 86 ^a^
DML-Agl-dA	47 ± 19 ^a^	55 ± 27 ^a^	23 ± 12 ^b^
O-Agl-dA	53 ± 27 ^a^	14 ± 15 ^b^	75 ± 59 ^a^
L-Agl-dA	31 ± 17 ^a^	15 ± 14 ^b^	21 ± 16 ^b^
O-Agl-A	36 ± 13 ^a^	10 ± 8 ^b^	46 ± 34 ^a^
L-Agl-A	11 ± 5 ^a^	7 ± 5 ^b^	10 ± 5 ^a,b^
α-tocopherol	378 ± 67 ^a^	243 ± 37 ^b^	279 ± 55 ^c^
γ-tocopherol	14 ± 8 ^a^	4 ± 2 ^b^	8 ± 2 ^c^

Abbreviations: Lignans: sum of pinoresinol and 1-acetoxy-pinoresinol; DMO-Agl-dA: decarboxymethyl oleuropein aglycone, dialdehyde form; DML-Agl-dA: decarboxymethyl ligstroside aglycone, dialdehyde form; O-Agl-dA: oleuropein aglycone, dialdehyde form; L-Agl-dA: ligstroside aglycone, dialdehyde form; O-Agl-A: oleuropein aglycone, aldehyde form; L-Agl-A: ligstroside aglycone, aldehyde form; β- and δ-tocopherol were not detected in any of the samples. The data are expressed as means ± standard deviations; *n* = 70; values in the same row with different superscript letters are statistically significantly different (*p* < 0.05).

**Table 3:** Sterol content (mg/kg) and composition, and erythrodiol and uvaol content (%) of the ‘Buga’, ‘Črnica’ and ‘Drobnica’ olive oilts.

Sterols	Unit	‘Buga’	‘Črnica’	‘Drobnica’	Purity Characteristics for Extra Virgin Olive Oil [7]
Cholesterol	%	0.09 ± 0.04 ^a^	0.13 ± 0.04 ^b^	0.09 ± 0.02 ^a^	≤0.5
24-methylene-cholesterol	%	0.19 ± 0.10 ^a^	0.08 ± 0.04 ^b^	0.18 ± 0.10 ^a^	
Campesterol	%	2.55 ± 0.18 ^a^	3.70 ± 0.29 ^b^	2.60 ± 0.16 ^a^	≤4.0
Campestanol	%	0.08 ± 0.03 ^a^	0.34 ± 0.08 ^b^	0.11 ± 0.05 ^a^	
Stigmasterol	%	0.78 ± 0.25 ^a^	1.00 ± 0.60 ^a^	0.75 ± 0.36 ^a^	
Clerosterol	%	1.06 ± 0.05 ^a^	1.05 ± 0.07 ^a^	0.99 ± 0.06 ^b^	
β-sitosterol	%	84.67 ± 1.99 ^a^	85.23 ± 1.90 ^a^	79.45 ± 4.03 ^b^	≥93.0
Sitostanol	%	1.04 ± 0.30 ^a^	3.51 ± 0.84 ^b^	1.94 ± 0.56 ^c^	
Δ-5-avenasterol	%	7.94 ± 1.84 ^a^	3.55 ± 1.74 ^b^	11.72 ± 4.15 ^c^	
Δ-5,24-stigmastadienol	%	0.85 ± 0.12 ^a^	0.63 ± 0.16 ^b^	1.26 ± 0.23 ^c^	
Δ-7-stigmastenol	%	0.20 ± 0.05 ^a^	0.29 ± 0.06 ^b^	0.24 ± 0.08 ^a^	≤0.5
Δ-7-avenasterol	%	0.57 ± 0.05 ^a^	0.49 ± 0.09 ^b^	0.68 ± 0.09 ^c^	
Apparent β-sitosterol	%	95.56 ± 0.45 ^a^	93.98 ± 0.56 ^a^	95.35 ± 0.36 ^a^	
Total sterols	mg/kg	2468 ± 251 ^a^	1351 ± 270 ^b^	2391 ± 314 ^a^	≥1000
Erythrodiol + uvaol	%	0.79 ± 0.33 ^a,b^	0.66 ± 0.25 ^a^	1.06 ± 0.75 ^b^	

Brassicasterol, Δ-7-campesterol and Δ-5,23-stigmastadienol were not detected in any of the samples. Erythrodiol and uvaol are expressed as a percentage of the total sterols. The data are expressed as means ± standard deviations; *n* = 46; values in the same row with different superscript letters are statistically significantly different (*p* < 0.05).

## Data Availability

All the data are published in this article.
